# Use of Surface Corrugations for Energy-Efficient Chaotic Stirring in Low Reynolds Number Flows

**DOI:** 10.1038/s41598-020-66800-5

**Published:** 2020-06-17

**Authors:** S. W. Gepner, J. M. Floryan

**Affiliations:** 1 Warsaw University of Technology, Institute of Aeronautics and Applied Mechanics, Nowowiejska 24, 00-665 Warsaw, Poland; 2 Department of Mechanical and Materials Engineering, The University of Western Ontario, London Ontario, N6A 5B9 Canada

**Keywords:** Physics, Applied physics, Fluid dynamics

## Abstract

We demonstrate that an intensive stirring can be achieved in laminar channel flows in a passive manner by utilizing the recently discovered instability waves which lead to chaotic particle movements. The stirring is suitable for mixtures made of delicate constituents prone to mechanical damage, such as bacteria and DNA samples, as collisions between the stream and both the bounding walls as well as mechanical mixing devices are avoided. Debris accumulation is prevented as no stagnant fluid zones are formed. Groove symmetries can be used to limit stirring to selected parts of the flow domain. The energy cost of flows with such stirring is either smaller or marginally larger than the energy cost of flows through smooth channels.

## Introduction

Mixing of fluids is a two-stage process composed of diffusion overlaid on top of mechanical stirring (advection)^1^. The former is dictated by material properties and is in general slow, while the latter is associated with flow kinematics. Stirring promotes mixing in the sense that it leads to stretching and folding^2,3^ of fluid interfaces, increasing concentration gradients and enabling the otherwise slow diffusion to act more rapidly and across shorter distances.

For the vector field to produce chaotic particle trajectories, the flow must be either unsteady two-dimensional or three-dimensional^4,5^. Turbulization provides chaotic motions but cannot always be achieved and comes with significant pressure losses. The first demonstration that chaos exists in laminar flows was provided by Arnold^6^ and Hénon^7^. The follow-up studies focused on “toy” flows whose forms are given analytically at the cost of truncated physics, e.g., ABC (Arnold-Beltrami-Childress) flows^8^, blinking vortex^9^, double gyre^10^ or the pulsed source-sink^11^, and provided fertile grounds for concept development^5^ and guidelines for creating real flows capable of chaotic stirring. Passive methods focus on geometry modifications where stirring is accomplished by forcing the flow to impact obstacles, e.g., partitioned pipe^12^, serpentine channel^13^, twisted pipe^14^, herringbone surface^15^ and various assemblies of bars and blades^16^ - stirring increases but at a cost of additional pressure losses. Active methods employ external body forces^17^, actuated particles^18^ and various stirrers^9^ with all of them requiring an external energy input. Three-dimensionality and unsteadiness do not guarantee chaos^19,20^, and not every chaotic state guarantees good stirring; either Poincaré maps or Lyapunov exponents must be used to verify whether the resulting flow is both chaos- and stirring-capable.

We report here the existence of flow systems where small geometry modifications create bifurcations resulting in a natural creation to new, chaos-capable states. These configurations are energetically efficient, i.e., maintaining the new flow either requires less energy than the reference flow or the energy consumption increases at a marginal rate. We focus on geometries that eliminate direct fluid collisions with the bounding walls in order to reduce mechanical damage to the mixture constituents and geometries that avoid formation of stagnant fluid zones so that any debris can be washed out by the stream. This leads us to analyze conduits with longitudinal grooves, which we divide into symmetry preserving grooves and symmetry breaking grooves. We demonstrate that separate stirring zones can be created in the former case without introducing any physical barriers.

## Geometry Modifications

**Figure 1:**
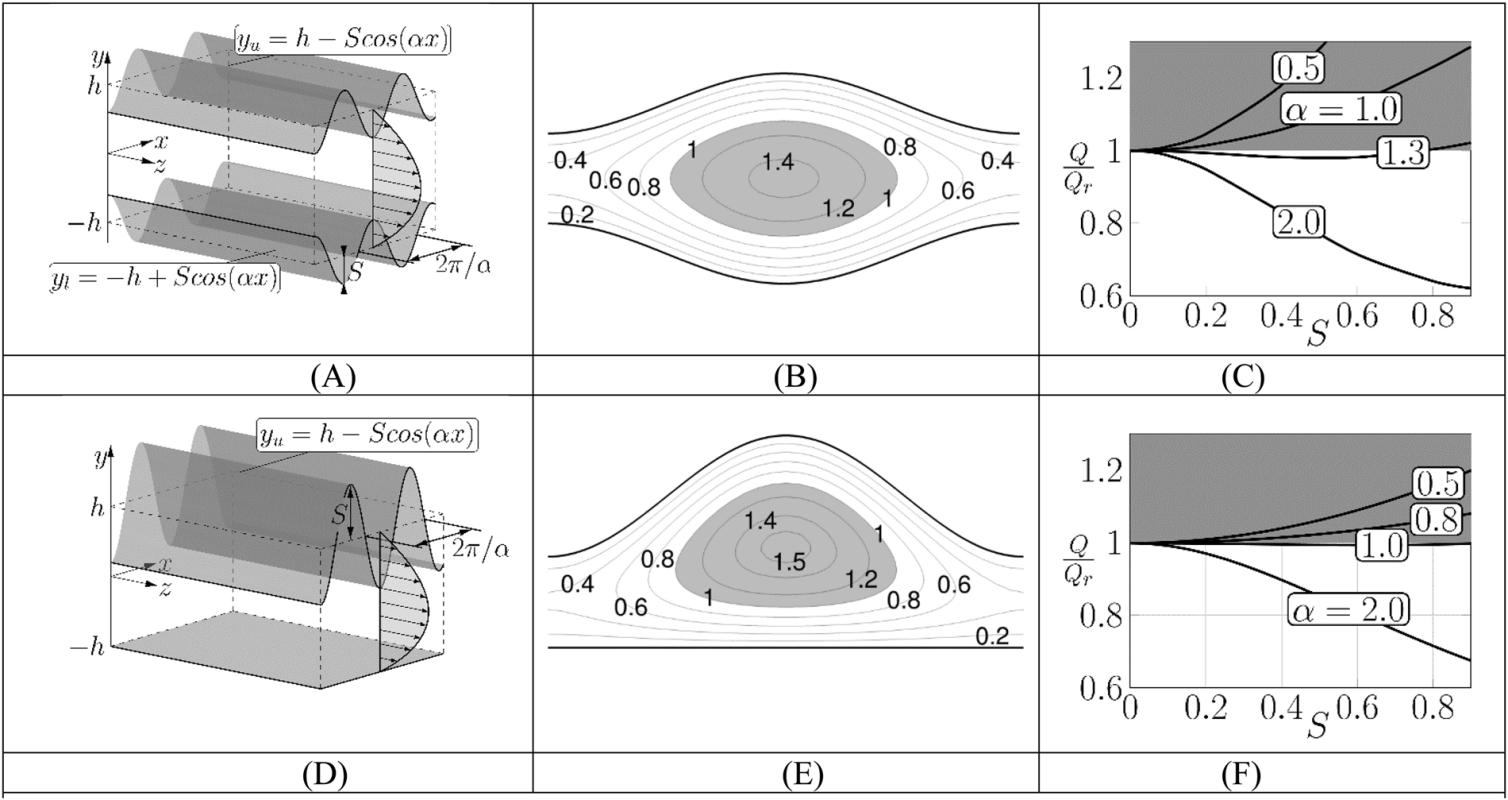
Primary flow. Figure 1A (1D) - symmetry preserving (breaking) grooves; Fig. 1B (1E) - contour plot of the streamwise velocity for the symmetry preserving (symmetry breaking) grooves for $S=0.4,\alpha =1$ *(S* = 0.8, *α* = 0.8). Grey color identifies zones of accelerated fluids. Figure 1C (1 F) - variations of the flow rate as a function of *S* and $\alpha$ for the symmetry preserving (symmetry breaking) grooves. Grey color identifies drag reducing configurations. *Q* (*Q*_*r*_) stands for the flow rate of the modified (unmodified) flow driven by the same pressure gradient.

We begin by defining the reference flow which we shall use to demonstrate that chaotic state is produced with less energy expenditures than those required to maintain the unmodified flow. The steady plane Poiseuille flow characterized by the Reynolds number *Re* = *W*_*max*_ *h*/*v* where *W*_*max*_ is the maximum of the streamwise velocity, *h* is the channel half-height and *v* is the kinematic viscosity, is chosen as the reference flow. It is driven by a constant pressure gradient *dp*/*dz* = −2/*Re* resulting in the velocity field $\overrightarrow{u}=[0,1,1-\,{y}^{2}]$ which produces the flow rate *Q*_*r*_ = 4/3. We now create spatial flow modulation by modifying the conduit geometry with grooves parallel to the flow direction (Fig. 1); we require that the mean channel cross section remains the same. We use Fourier expansions to characterize geometric patterns with commensurability factors (ratios of various geometric wave numbers) providing the means to create flow adjustments. Grooves on both walls lead to a pattern interaction problem providing additional degrees of freedom. The analysis relies on numerical solutions utilizing finite-length computer words which precludes access to non-commensurable topographies. Since various features of the self-induced chaotic stirring can be demonstrated using very simple geometries, we defer the above complexities to later analyses and focus the discussion on grooves described by a single Fourier mode. For detailed discussion, we select conduits with either symmetry-preserving (Fig. 1A) or with symmetry-breaking (Fig. 1D) grooves. The modified flow is steady, one-dimensional and *Re*-independent as scaling eliminates *Re* (see the methodology section) without any promise for chaotic motions. Typical flow topologies (see Fig. 1B,E) demonstrate the formation of high-velocity stream tubes located in the widest channel openings where the fluid accelerates beyond the maximum velocity of the reference flow. Conduits with grooves of sufficiently long wavelengths are more energy efficient in the sense that the same pressure gradient generates a larger flow rate than in the unmodified conduit^21^ (see Fig. 1C,F) with the symmetry-preserving grooves being more efficient than the similar symmetry-breaking grooves. The most energy efficient grooves have universal constraint-dependent shapes, e.g., golden trapezoid, Gaussian function^22,23^.

## Flow bifurcation

**Figure 2:**
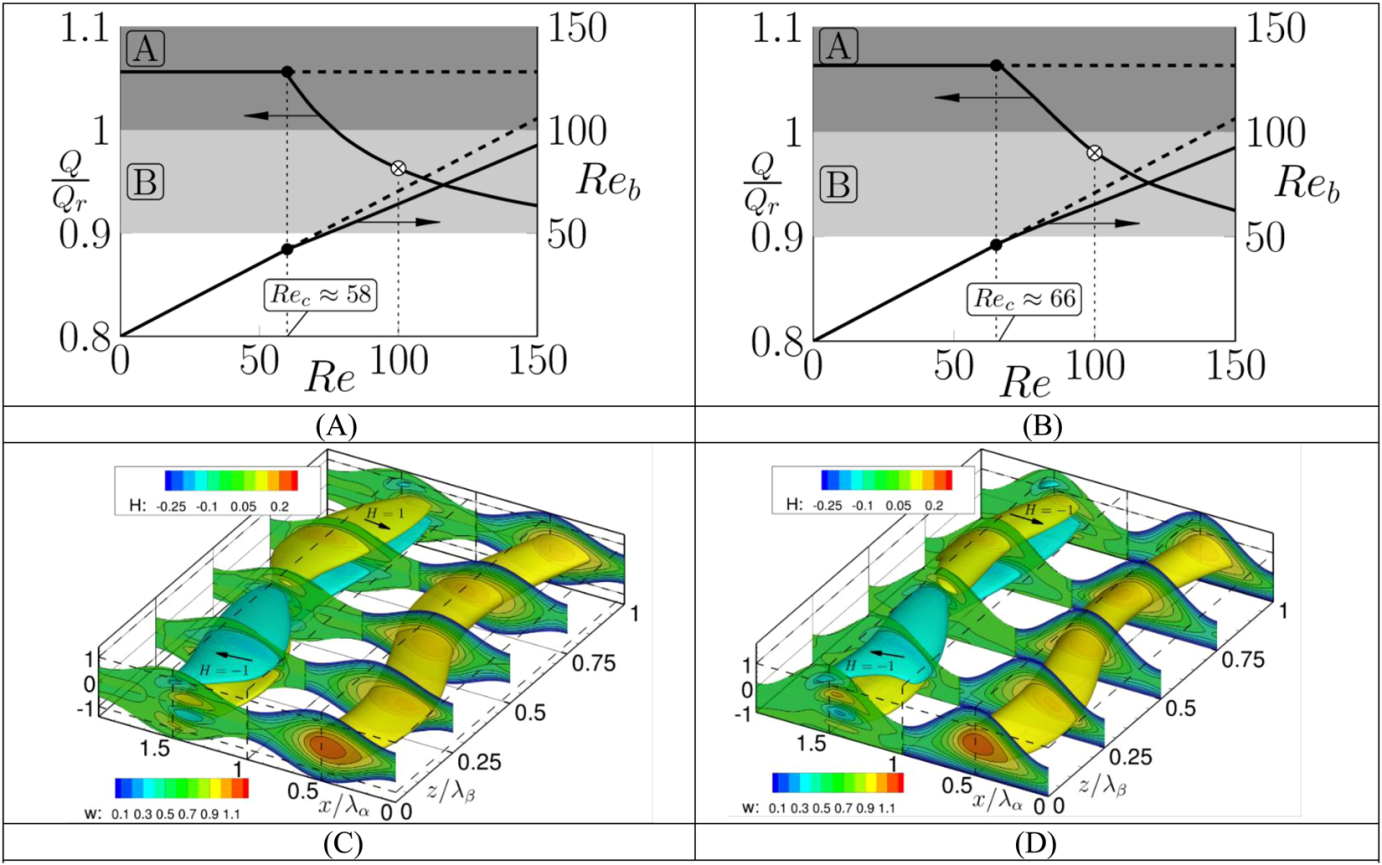
Bifurcation diagrams and instantaneous flow fields for the symmetry-preserving (Fig. 2A,C; ($\alpha ,S,\beta \,=$ (1, 0.4, 0.4)) and symmetry-breaking (Fig. 2B,D; ($\alpha ,S,\beta \,=$ (0.8, 0.8, 0.3)) grooves. *Q* stands for the flow rate of the modified flow, *Re* is the Reynolds number of the reference flow and *Re*_*b*_ is the bulk Reynolds number of the modified flow based on the average streamwise velocity. Zones *A* in Fig. 2A,B identify conditions leading to the reduction of pressure losses and zones *B* identify conditions leading to an increase of pressure losses by up to 10% of the reference losses. Solid dots identify bifurcation points while crossed dots identify conditions used in Fig. 2C,D. The left sides of Fig. 2C,D display helicity *H* defined as $H=\overrightarrow{u}\cdot (\nabla \times \overrightarrow{u})$ with arrows showing the direction of the instantaneous rotation while the right sides display contour plots of the stream*w*ise velocity component *w* (yellow color identifies stream tube with $w\ge 1$). *λ*_*α*_ is the groove wavelength while *λ*_*β*_ is the wave wavelength.

The first step towards flow evolution to a chaotic state is the onset of the recently discovered inviscid^24^ instability modes with the critical *Re* being as low as O(10^2^)^24–26^. We illustrate in Fig. 2A,B the formation of flow bifurcations with different classes of grooves having the same effective amplitude (ratio of the widest and narrowest channel openings) to facilitate meaningful comparisons. The dark grey zones identify conditions leading to a reduction of pressure losses while the light grey zones illustrate conditions leading to a small increase of losses, which we define as losses of up to 10% of the reference flow rate. We use the linear stability theory to determine the critical conditions, which are *R*_*cr*_ = 58.8, *β*_*cr*_ = 0.4, *σ*_*cr*_ = (0.334, 0) for the conditions used in Fig. 2A, and *R*_*cr*_ = 65.43, *β*_*cr*_ = 0.3, *σ*_*cr*_ = (0.315, 0) for the conditions used in Fig. 2B. In the above, $\beta$ stands for the streamwise disturbance wave number, the real part of $\sigma$ stands for the frequency and its imaginary part describes the amplification rate. The flow resistance increases (and the flow rate decreases) as the system moves along a bifurcation branch towards higher *Re* as the same pressure gradient drives a more complex flow.

We determine properties of saturation states through time integration of the complete flow equations as explained in the Methodology section. When *Re* is small enough (*Re* < ~80), the modified conduits produce a larger flow rate than the reference conduit (Fig. 2A,B). The saturation states have the form of high velocity stream tubes modulated by instability waves (see the right sides of Fig. 2C,D and supplementary videos *S*1 and *S*2) producing a three-dimensional time-dependent flow. This flow contains zones of fluid rotating in opposing directions above and below the stream tube (see helicity plots on the left sides of Fig. 2C,D) with the direction of rotation changing every half wavelength with a small overlapping zone containing four layers of counter-rotating fluid. As a result, fluid particles moving downstream experience periodic changes in the direction of rotation, which is not unlike the effects created by the blinking vortex^9^, double gyre^10^, pulsed source-sink^11^, partitioned pipe^12^, serpentine channel^13^, twisted pipe^14^ and herringbone surface^15^.

## Chaotic states

**Figure 3:**
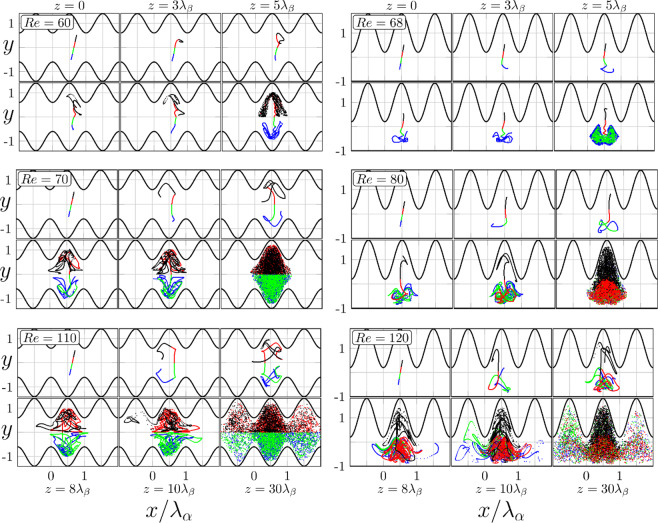
Locations of particles in the (*x*,*y*)-planes at different *z*-locations. Blue and green (red and black) colors identify particles placed below (above) the line *y* = 0. The left (right) column refers to the symmetry preserving (breaking) grooves.

**Figure 4:**
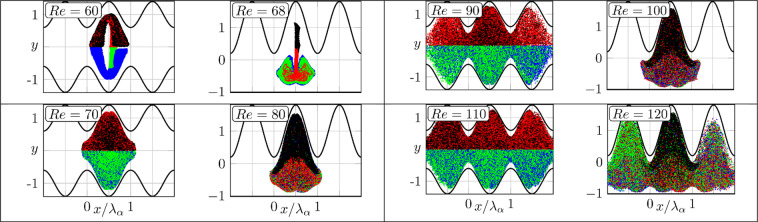
Poincaré sections formed by particles’ trajectories between *z* = 0 and *z* = 50 *λ*_*β*_. The initial particle positions and colors are the same as in Fig. 3. The images were produced by displaying non-transparent particles in the following order: blue, green, red, black.

We shall now demonstrate the formation of chaotic states by examining the advection of massless particles. We place them in the flow at *z* = 0 along a straight line extending between (*x,y*) = (3.5,−0.5) and (4.5,0.5) and follow their trajectories. We tested different placement times (instantaneous versus distributed) and different initial positions and concluded that the process is ergodic and, therefore, dependence on the initial conditions averages out for long enough tracings. Intersections of trajectories with transverse planes at different *z*-locations are shown in Fig. 3; the images are organized in pairs of *Re*’s equally distant from the bifurcation points for both types of grooves. The initially straight material lines loose coherence within approximately five wave wavelengths in the streamwise direction; this is expected as these lines are made of a finite number of particles. The stirring process is more rapid for larger *Re*’s which is also expected. In the case of symmetry-preserving grooves, the flow field is divided along the line of symmetry into two zones with no advective transport taking place across the separatrix for all *Re*’s considered. In the case of symmetry-breaking grooves, advection (understood as wandering of trajectories) occurs in the whole domain. It is remarkable that particles spread in the spanwise direction well beyond the groove segment where they were initially placed, i.e., chaotic movement does not obey the spanwise periodicity condition, with this effect being more pronounced for the symmetry-breaking grooves. Poincaré sections displayed in Fig. 4 demonstrate the ability of the flow to promote particle spreading as, when time progresses, an increasing portion of the domain is visited by trajectories originating from the same initial set except for regions near the walls, which is entirely expected. We did not find any elliptic islands^12,27^ trapping the fluid for the conditions considered in this study.

**Figure 5:**
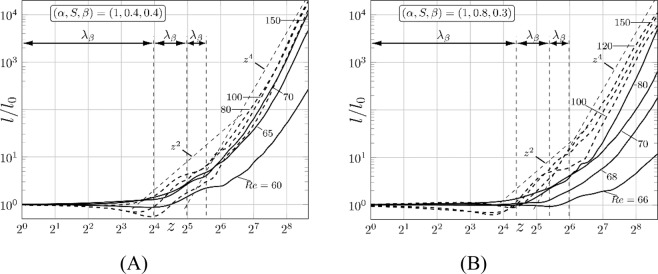
Stretching of material lines used in Fig. 3 as functions of the *z*-distance from their initial placement for the symmetry preserving (Fig. 5A) and the symmetry breaking (Fig. 5B) grooves. The solid and dashed lines correspond to the drag reducing and slightly drag increasing (10% increase) flow conditions. Horizontal axes end at *z* = 400.

To quantify the ability of the flow field in creating folding and stretching of material lines, we measure the length of an initially straight material line by following the trajectories of its elements until they intersect with a transverse plane (*z* = const), and measure the length *l* of the lines formed by the intersection points – this length is defined as the Euclidian distance between the consecutive points. The initial length is *l*_0_ at *z* = 0 and the ratio *l*/*l*_0_ provides a measure of stretching. In the case of unmodified flow, *l* = *l*_0_ for all times as particles follow straight trajectories always producing the same projections. These projections are not affected by the streamwise spreading due to flow shear. In the case of modified flows, projections capture spanwise spreading which is entirely due to chaotic mixing and begins to be observable at approximately one wave wavelength downstream from the particle insertion point (Fig. 5A,B). The stretching increases initially proportionally to *z*^2^ and then proportionally to *z*^4^ for all *Re’*s used in this study, with the growth rate reaching saturation more rapidly for higher *Re* for both types of grooves (Fig. 5).

## Stirring quantification

**Figure 6:**
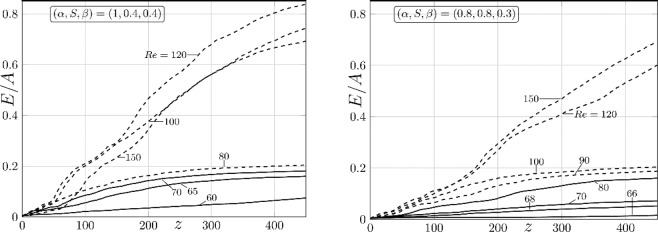
Variations of the *E/A* measure of mixing intensity as a function of the *z*-distance from the particle injection plane. The left (right) column refers to the symmetry-preserving (breaking) grooves. The solid and dashed lines correspond to the drag reducing and slightly drag increasing (10% increase) conditions.

We shall use two different measures for stirring quantification. In the first one, we introduce 100 × 100 approximately equal bins uniformly distributed across five adjacent groove sections $[z\in (-2{\lambda }_{\alpha },3{\lambda }_{\alpha })]$ and define the *E/A* measure as the ratio of the ever-occupied bins up to a given *z*-location to the total number of bins. Each bin is turned on when at least one particle is found and remains turned on regardless whether the particle remains or leaves – it can be interpreted as a quantification of Poincare sections. The particle spreading increases with distance, eventually approaching a *Re*-dependent saturation state (Fig. 6) which corresponds to *E/A* of up to 20% for the drag reducing configurations and *E/A* of up to 80% for the configuration resulting in a slight increase of losses.

**Figure 7:**
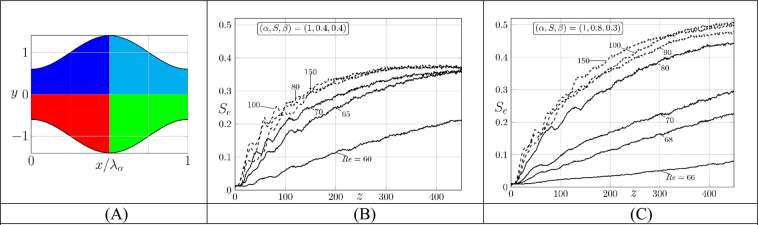
The initial distribution of four distinct species is illustrated in Fig. 7A for the symmetry-preserving grooves. Variations of the Shannon entropy *S*_*e*_ as a function of the *z*-distance from the initial particles’ placement for the symmetry-preserving (Fig. 7B) and the symmetry-breaking (Fig. 7C) grooves. The solid and dashed lines correspond to the drag reducing and slightly drag increasing (10% increase) conditions.

The second measure uses Shannon entropy^27,27^. We measure the order of the system in terms of the probability of finding a given state. Entropy takes low values when the probability of selecting one of the available states is much higher than that for the others. In stirring, low values correspond to different constituents being separated so that the presence (absence) of one of them can be easily predicted. High values correspond to well-stirred states when identification of different components becomes difficult. We divide the domain into $i=1,2,\ldots ,M$ “bins” and introduce $i=1,2,\ldots ,C$ species (particles of different colors). The probability of finding particles of a given type in bin *j* is ${p}_{j}(i)={n}_{i,j}/{n}_{j}$, where ${n}_{i,j}$ is the number of particles of type *i* in bin *j* and ${n}_{j}$ is the total number of particles in bin *j*. The entropy for bin *j* is ${S}_{j}=-\,{\sum }_{i=1}^{C}{p}_{j}(i)log[{p}_{j}(i)]$ and the overall entropy averaged over all bins is $S={M}^{-1}{\sum }_{j=1}^{M}{S}_{j}$. The results are normalized with the maximum possible entropy ${S}_{max}$ which corresponds to a perfectly stirred state where the probability of finding a particle of each type in each bin is the same, i.e., ${p}_{j}(i)=1/C$. This leads to ${S}_{max}=-\,{M}^{-1}{\sum }_{j=1}^{M}{C}^{-1}log({C}^{-1})$, and the Shannon entropy is expressed as ${S}_{e}=S/{S}_{max}$ with ${S}_{e}\le 1$. We use the test domain consisting of one groove segment combined with spanwise periodicity for the particle movement between grooves which means that the total number of particles in the test section remains the same. We sub-divide the test domain into 100 × 100 approximately equal bins, organize them into four zones, fill each zone at *z* = 0 with a uniform distribution of distinct species (Fig. 7A) and follow particles’ movement in the downstream direction. *S*_*e*_ increases with *z* and reaches a saturation within approximately 15 wave wavelengths from the particles injection point – this saturation determines the maximum possible stirring which has been found to correspond to ${S}_{e}\approx 0.4$ for the symmetry-preserving grooves and ${S}_{e}\approx 0.5$ for the symmetry-breaking grooves (Fig. 7B,C). Stirring very closed to its maximum can be achieved with reduction of pressure losses below those associated with the simple, unstirred reference flow. The difference between *S*_*e*_’s observed for both types of grooves can be explained by noting that the flow domain is divided into two separate stirring zones in the symmetric channel (see Figs. 3–4). This means that only two species mix in each of the upper/lower portions of the channel and, since the required travel distances are smaller, the final state is achieved faster. *S*_*e*_ is the same for each portion but should be halved if a complete channel is considered. The stirring occurs in the whole channel for the symmetry-breaking grooves, the particles must travel over longer distances and, as a result, the best possible mixed states are achieved further downstream. These states are however characterized by larger *S*_*e*_’s than those achieved using the symmetry-preserving grooves (Figs. 3, 5 and 7).

## Conclusions

We have demonstrated that the use of properly shaped grooves reduces flow losses and forces the flow to bifurcate to a form which produces chaotic states. The flow field has a regular time- and spatially- periodic structure but it generates chaotic particle trajectories. The chaotic character of the trajectories was confirmed through evaluation of folding and stretching of the test material lines, and through construction of Poincaré sections. The resulting stirring has been quantified using two measures. In the first one, test particles were injected into the flow field which was divided into a system of small bins and the ratio of the ever-occupied bins to the total number of bins was determined. The second measure used the Shannon entropy concept. The stirring can occur either in the complete flow domain or in different subdomains depending on the system symmetries – no physical barrier is required to separate stirring subdomains in the latter case. The energy cost of such stirring can be smaller than the energy cost of the reference flow without any stirring.

## Methodology

We solve the incompressible Navier-Stokes equations $\frac{\partial \overrightarrow{u}}{\partial t}+\overrightarrow{u}\cdot \nabla \overrightarrow{u}=-\,\overrightarrow{\nabla p}+{R}^{-1}\Delta \overrightarrow{u},\cdots \nabla \cdot \overrightarrow{u}=0$ with $(u,v,w)$ being the spanwise *x*-, normal-to-the-wall *y*- and streamwise *z*- components of the velocity vector, and *p* denoting pressure. The flow is driven by a constant pressure gradient applied along the *z*-direction. The channel geometry for the symmetry-breaking grooves is given as ${y}_{L}(x,z)=-\,1+Scos(\alpha x),{y}_{U}(x,z)=1$ and for the symmetry-preserving grooves as ${y}_{L}(x,z)=-\,1+Scos(\alpha x),{y}_{U}(x,z)=1-Scos(\alpha x)$ where *S* and *α* stand for the groove amplitude and wavenumber, respectively.

The field equations for the primary state reduce to a single equation of the form $\frac{{\partial }^{2}w}{\partial {x}^{2}}+\frac{{\partial }^{2}w}{\partial {y}^{2}}=-\,2$ which was discretized using the spectral element method^28^ with the resulting linear algebraic equations solved using standard solvers.

The stability of the primary state was analyzed using temporal linear stability in the asymptotic (modal) formulation. Disturbances were assumed to be in the form ${\overrightarrow{u}}_{d}(x,y,z,t)={\overrightarrow{u}}_{a}(x,y){e}^{i(\delta x+\beta z-\sigma t)}+CC$ where ${\overrightarrow{u}}_{d}=({u}_{d},{v}_{d},{w}_{d})$ is the disturbance velocity vector, ${\overrightarrow{u}}_{a}(x,y)$ is the amplitude function vector, $\delta$ is the spanwise wave number, $\beta$ is the streamwise wave number, $\sigma$ is the complex amplification rate and *CC* stands for the complex conjugates. The spectral element discretization^28^ was used to convert the resulting eigenvalue problem for the partial differential equations for the modal functions into an algebraic eigenvalue problem which was then solved using standard methods^24,25^.

Nonlinear saturation states were obtained by direct numerical time integration of the full field equations using the spectral elements in the spanwise *x*- and normal-to-the-wall *y*-directions and Fourier decomposition in the *z*-direction^29^ combined with the second-order velocity-correction scheme^30^. A regular, structured mesh made of quadrilateral elements was generated using the GMSH package^31^. We used 12 elements in the *x*-direction and 10 in the *y*-direction per single corrugation. Spectral discretization within each element used nine modified Jacobi polynomials and nine Gauss-Lobatto-Legendre quadrature points in each direction^29^. We used NEKTAR++ implementations^32^ of these methods in our work.

The numerical error is dictated by several factors. The Fourier expansion was truncated after *M* modes with *M* selected to make ratio of kinetic energies of this mode and mode zero small enough (10^−20^ was used in the computations). The spectral element mesh as well as the local expansions were selected based on the convergence studies carried out previously in the context of stability and nonlinear saturation studies^19,26,33^. Sufficient temporal accuracy and resolution were achieved with the step size of $\Delta t=$ 2e-2^26,33^, which translates to approximately 1000 timesteps per period of the oscillatory flow. The computational box extended over two groove wavelengths in the spanwise direction and over a single wavelength of the travelling wave in the streamwise directions to account for possible *x*- and *z*-subharmonics, but none were found. The adequacy of the box size was tested by repeating certain cases with doubling of its size and no differences within an acceptable numerical error were found.

Lagrangian particle tracking relied on numerical solution of $\frac{d\overrightarrow{x}}{dt}=\overrightarrow{u}(\overrightarrow{x},t)$ which was carried out using a 4^th^-order Runge-Kutta method. Here $\overrightarrow{x}$ is the position vector and $\overrightarrow{u}(\overrightarrow{x},t)$ is the instantaneous velocity vector. The error in tracking of material lines was controlled through the use of a large number of material points and by limiting the length of simulation time interval.

## Supplementary information

Supplementary Information.

Supplementary Information 2.

Supplementary Information 3.
