# Supplementary material for: Use of Surface Corrugations for Energy-Efficient Chaotic Stirring in Low Reynolds Number Flows
Source: Sci Rep. 2020 Jun 17;10:9865. doi: 10.1038/s41598-020-66800-5 (PMC7300130; doi:10.1038/s41598-020-66800-5)
Supplement: Supplementary file 1 — Supplementary Information. [file 41598_2020_66800_MOESM1_ESM.pdf]

Non-Intrusive Energy-Efficient Chaotic Stirring

S. W. Gepner[1*] and J. M. Floryan[2]

Guide to supplementary materials

The supplementary material consists of two animations illustrating flow evolution resulting from amplification of instability waves. Left sides display helicity $H = \vec{u} \cdot (\nabla \times \vec{u})$ which illustrate fluid rotation. Right sides display streamline velocity component which illustrate formation of high velocity stream tubes and their flexing due to instability. Inserts show the in-plane motion when a vortical structure passes through the test plane.

topology_onesided_a0.8_Re=100.avi:
Authors: S. W. Gepner and J. M. Floryan
Flow evolution in the case of symmetry-breaking grooves for ($\alpha, S, \beta =$(0.8, 0.8, 0.3)).

topology_twosided_a1_Re=100.avi:
Authors: S. W. Gepner and J. M. Floryan
Flow evolution in the symmetry-preserving grooves for ($\alpha, S, \beta =$(1.0, 0.4, 0.4)).
